# Gut Microbiota and Time-Restricted Feeding/Eating: A Targeted Biomarker and Approach in Precision Nutrition

**DOI:** 10.3390/nu15020259

**Published:** 2023-01-04

**Authors:** Falak Zeb, Tareq Osaili, Reyad Shakir Obaid, Farah Naja, Hadia Radwan, Leila Cheikh Ismail, Hayder Hasan, Mona Hashim, Iftikhar Alam, Bismillah Sehar, MoezAllslam Ezzat Faris

**Affiliations:** 1 Research Institute for Medical and Health Sciences, University of Sharjah, Sharjah 27272, United Arab Emirates; 2 Department of Clinical Nutrition and Dietetics, College of Health Sciences, University of Sharjah, Sharjah 27272, United Arab Emirates; 3 Department of Nutrition and Food Technology, Faculty of Agriculture, Jordan University of Science and Technology, P.O. Box 3030, Irbid 22110, Jordan; 4 Department of Human Nutrition and Dietetics, Bacha Khan University Charsadda, Peshawar 24540, KP, Pakistan; 5 Department of Health and Social Sciences, University of Bedfordshire, Luton LU1 3JU, UK

**Keywords:** gut microbiome, time-restricted feeding, intermittent fasting, targeted approach, hormonal signaling, metabolic regulators

## Abstract

Each individual has a unique gut microbiota; therefore, the genes in our microbiome outnumber the genes in our genome by about 150 to 1. Perturbation in host nutritional status influences gut microbiome composition and vice versa. The gut microbiome can help in producing vitamins, hormones, and other active metabolites that support the immune system; harvest energy from food; aid in digestion; protect against pathogens; improve gut transit and function; send signals to the brain and other organs; oscillate the circadian rhythm; and coordinate with the host metabolism through multiple cellular pathways. Gut microbiota can be influenced by host genetics, medications, diet, and lifestyle factors from preterm to aging. Aligning with precision nutrition, identifying a personalized microbiome mandates the provision of the right nutrients at the right time to the right patient. Thus, before prescribing a personalized treatment, it is crucial to monitor and count the gut flora as a focused biomarker. Many nutritional approaches that have been developed help in maintaining and restoring an optimal microbiome such as specific diet therapy, nutrition interventions, and customized eating patterns. One of these approaches is time-restricted feeding/eating (TRF/E), a type of intermittent fasting (IF) in which a subject abstains from food intake for a specific time window. Such a dietary modification might alter and restore the gut microbiome for proper alignment of cellular and molecular pathways throughout the lifespan. In this review, we have highlighted that the gut microbiota would be a targeted biomarker and TRF/E would be a targeted approach for restoring the gut-microbiome-associated molecular pathways such as hormonal signaling, the circadian system, metabolic regulators, neural responses, and immune-inflammatory pathways. Consequently, modulation of the gut microbiota through TRF/E could contribute to proper utilization and availability of the nutrients and in this way confer protection against diseases for harnessing personalized nutrition approaches to improve human health.

## 1. Introduction

Microbiota in the gastrointestinal tract is seeded just after birth. In the complete life span of the human being, more than 60 tons of food passes through the entire gastrointestinal tract of 250–400 m^2^ [1], due to which bacteria enter the body, some of which get colonized in the tract. These colonized collections are called gut microbiota [2]. The makeup of the gut microbiota can change over a person’s lifetime, despite the fact that it is subject to the combined influence of host genetics and environmental factors. The microbial community’s nature, remodeling, and responses to dietary nutrients are all influenced by the host’s genetic background [3]. These changes can occur in the gut microbiome and host nutritional status. Fluctuations in the microbiota have been associated with the emergence of metabolic syndrome, which encompasses diabetes and obesity. On the other hand, the gut microbiota also has an advantageous role in the human body. The main benefits are its roles in the integrity of the mucosal barrier; synthesis of the essential vitamins including vitamin K, biotin, nicotinic acid, riboflavin, pyridoxine, pantothenic acid, and thiamine for the protection against infected agents; boosting the immune system; training the circadian rhythm; and the neurological function of the body [3].

Similarly, gut microbiota act as a factory of substances that affect the chronobiology, metabolic sensors, inflammatory cytokines, neurological function, and the immune system. Intestinal microbes consume nutrients from the meal to produce energy and metabolites. Many of these metabolites are subsequently taken into circulation, where they may go through extra metabolism and change the host metabolism and nutritional status. The impact of bacterial metabolites on the metabolism of the host might occasionally be detrimental. For instance, short-chain fatty acids (SCFAs), which are produced from ordinarily indigestible fiber, have effects that are generally advantageous for the host. These effects include activities that fight against obesity and diabetes [4]. On the other side, N-nitroso compounds, ammonia, and hydrogen sulfide created by bacteria from dietary protein can generate reactive oxygen species (ROS) and precipitate in DNA damage. These compounds can also activate pro-inflammatory pathways. The end product of dietary choline known as trimethylamine-N-oxide (TMAO) promotes the development of atherosclerosis and has a correlation with cardiovascular disease (CVD), stroke, and mortality [3]. Numerous metabolites produced in the gut enter the bloodstream and can either act immediately or undergo additional metabolism by the host, producing bioactive chemicals that might affect the host’s metabolism and tissue function. In addition to facilitating fat absorption, the secondary bile acids are also reabsorbed into the bloodstream, where they act as ligands for the host cells’ farnesoid X (FXR) and TGR5 bile acid receptors, having impacts such as on the immunological function and energy metabolism. Similarly, SCFAs made by bacteria, such as acetate, butyrate, and propionate, not only serve as vital energy sources for the liver and intestinal epithelium but also have the power to alter insulin secretion, immune system activity, appetite, brain function, and adipose tissue. These SCFAs influence the immune, hormonal, and neurological systems of the body as they are involved in the production of cytokines, chemotaxis, neurotransmitters, endocrine signals, and the apoptosis process [3,4]. As a result, these modifications of the microbiome communities in the gut have the potential to play a part in the emergence of metabolic illnesses such as type 2 diabetes, weight gain, and metabolic syndrome.

Interestingly, a variety of classes of gut bacterial composition and metabolites can be affected by necessities such as food, nutrition composition, and patterns of eating and fasting. Time-restricted feeding/eating (TRF/E) is one of the most striking eating patterns that had been followed by humans for the last many years and has demonstrated benefits independent of energy restriction in both animals and humans [5]. This pattern allows no caloric restriction and has an eating window period of as much as 10 h in a day [6]. Many recent studies have demonstrated that TRE has a great impact on gut microbiota composition and showed that time restriction and meal sizes change the proportions and abundance of microbiota [7]. Similarly, our recent studies showed that TRE increases microbial richness and diversity in healthy male adults [8], and polyunsaturated fatty acids (PUFA), vitamin D, iodine, vitamin E, magnesium, and carbohydrates were in abundance in the gut of TRF practicing groups [9]. Precision nutrition provides tailored dietary interventions and recommendation based on an individual genetic makeup, metabolic profile, and environmental exposure [10]. Depending on an individual’s genetic makeup, nutrigenomics workhorse of precision nutrition may evaluate the different phenotypic responses to specific diets [11]. Precision nutrition may integrate the information about microbiota and adding dietary challenges to magnify the interindividual differences in postprandial response. Personalized gut microbiome features might help predict an individual postprandial glycemic response to dietary components [12,13]. The basic gut microbial profile may be a predictor for an individual’s response to dietary interventions. Fermentation products of carbohydrates and proteins induce the microbiome-associated effects on host metabolism [14]. However, the use of the gut microbiome as a biomarker to predict responsiveness to specific dietary ingredients to develop precision diets and interventions [15] is very important for optimal health. Therefore, it is very important to adopt an accessible and non-invasive approach to better tackle the biomarkers of many metabolic diseases in the shape of healthy gut microbiota.

## 2. Gut Microbiota: A Targeted Biomarker

Greater microbiota diversity is associated with improved lipid profiles, anti-inflammatory cytokines, liver enzymes, and eventually genetic pathways, all of which are metabolic indications for better health [8,16]. Prior research has demonstrated that Sirt1 is a crucial regulator and promoter for the production of clock genes, as well as establishing a biological link between the control of metabolism and circadian rhythms [17,18]. There is mounting proof that the gut microbiota composition is associated with Sirt1 expression and proportionally affects the brain via neurological, endocrine, and immunological channels. The gut microbiome makeup is of particular interest when it comes to cognition and brain-related disorders [19]. Both the abnormal gut flora and the daily cycle of feeding/fasting have an impact on the host metabolism and aid in the emergence of metabolic diseases such as obesity. It is believed that eating and fasting cycles cause periodic changes in the gut microbiome, which act as a mechanism for controlling host metabolism. These differences add to the variety of gut microflora. Therefore, the feeding pattern, time, and length, as well as the composition of the meal, are significant characteristics to consider when determining the contribution of the microbiome to the physiology and metabolism of the host [20]. Communication between the gut and the brain is essential for determining the appropriate portion size of a meal and sending signals to the brain to control feelings of hunger and fullness. Mechanosensitive gastric vagal afferents (GVAs) display diurnal rhythmicity in the nutrient composition and chemical pathways in response to food-related stimuli. This allows for satiety signaling to occur at a specific time of day through gut–brain communication [21]. As a result, the absence of a diurnal rhythm in the GVA axis can contribute to an increase in both hyperphagia and obesity. Recent studies on both animals and humans have revealed that the emergence of obesity is correlated with a lower microbiota diversity, changed gut microbiota activity, and dispersed microbiota abundance, specifically of two phyla, namely, *Bacteroidetes* and *Firmicutes* [22]. When *Bacteroidetes* are allowed to remain in the gut, they continue to maintain a dynamic and, for the most part, beneficial relationship with the host [23].

Gut microbiota dysbiosis is a mediator for the emergence of several human illnesses [24]. It was shown that the prevalence of metabolic and inflammatory diseases such as obesity, atherosclerosis, neurological disorders, and diabetes correlates inversely with the number of *Bacteriodetes* [25]. Similarly, *Bacteroidia* was found to have an inverse correlation with low-density lipoprotein (LDL) and triglyceride (TG) levels, and both types of these bacteria exhibited an anti-obesity response. In a similar pattern, a drop in mouse body weight is closely correlated with an increase in members of the genus *Bacteriodetes* [26]. The dysbiosis-induced circadian misalignment and other disturbed host–microbe interactions may contribute to the etiology of metabolic diseases. In ApoE-/- mice, overexpression of Bmal1 modifies lipoprotein synthesis and biliary cholesterol excretion, which lowers hyperlipidemia and atherosclerosis [27]. In addition to the timing of meals and the length of daylight, microbiota play a role in the control of a circadian system that is responsible for the regulation of intestinal physiology and systemic metabolism [28]. The circadian rhythmicity of the gut microbiome contributes to the proper functioning of the circadian clock of the host. Recent research conducted by our team revealed a substantial encouraging association between Bmal1 and *Prevotella* and *Bacteroidia*, as well as between Sirt1 and *Prevotellaceae*, *Bacteroidia*, and *Dialisster* [8]. In mouse tissues, the peripheral clock can be adjusted more easily through SCFAs derived from *Prevotella* that were produced by the fermentation of non-digestible fiber [29]. During the process of gut microbiome ablation, Bmal1 expression becomes disrupted, leading to a pre-diabetic phenotype and increased ileal corticosterone production. Inadequate levels of healthy gut microbiota also contribute to a general downward trend in the expression of clock control genes, which are particularly involved in the regulation of metabolic processes [30].

### 2.1. *Gut Microbiota and Host Energy Homeostasis*

At this time, intestinal microbiota can have a significant impact on the metabolic pathways involved in energy production in both human and animal models through pleiotropic mechanisms. Clinical investigations have revealed that atypical antipsychotic medications (AAPDs) may cause metabolic abnormalities with lower energy expenditure and body weight gain caused by gut microbial dysbiosis [31]. Through two complementary yet distinct pathways that result in a reduction in fatty acid metabolism, the gut microbiota may have an impact on obesity. These mechanisms are (i) reduced levels of the fasting-induced adipose factor (Fiaf), which inhibits the production of the peroxisomal proliferator-activated receptor co-activator (PGC-1), and (ii) decreased AMP-activated protein kinase (AMPK) activity of the liver and muscle. These findings lend credence to the idea that gut bacteria can influence both the demand and supply ends of the energy balance equation. In other words, the gut microbiota influences both the regulation of energy consumption and storage, as well as the harvesting of energy from the diet [32].

### 2.2. Gut Microbiota and Hormonal Signaling

Through the growth hormone secretagogue receptor 1 (GHS-R1a), the orexigenic hormone ghrelin regulates body weight [33]. Regardless of the fact that they alter lipid and glucose metabolism [34], the levels of the hormone ghrelin have been shown to increase in patients who are receiving treatment with AAPDs, according to clinical observations [35]. Recent research has shown that live microorganisms can influence the ghrelin system by modulating the GHS-R1a receptor. Furthermore, it has been established that several strains of *Lactobacillus* and *Bifidobacterium* had the same capacity to modify the ghrelin receptors. Therefore, blocking ghrelin signaling through a gut-microbiota-assisted plan may be encouraging treatment options to aid overweight patients who have been caused to gain weight by AAPDs for maintaining their weight loss [29].

Cholecystokinin, glucagon-like peptide-1 (GLP-1), 5-hydroxytryptamine, peptide YY (PYY), and leptin are all examples of hormones that participate in hormonal signaling. All of these hormones are crucial for controlling metabolic processes including hunger, fat storage, and the metabolism of glucose and lipids [36,37,38]. Microbiota such as *Oscillibacter* spp. and *Lactobacillus* spp. can influence the secretion of hormones such as PYY and GLP-1, and as a result, microbially mediated gut hormone participates in the regulation of host metabolism [38,39]. Satiety peptides PYY, GLP-1, and cholecystokinin were expressed less strongly in germ-free mice, hypothesizing that the gut microbiota may be responsible for stimulating the production of these hormones [40]. Furthermore, *E. coli* in the microbiome may boost enteroendocrine cells’ production of GLP-1 and PYY [41]. Products of bacteria, such as SCFAs, regulate the release or production of anorexic hormones (PYY and GLP-1), and they do this by binding to free fatty acid receptors (FFAR) 2 and FFAR 3 [42], possibly resulting in obesity. Nurmi and colleagues presented evidence that the microbes in the gut are responsible for the weight gain that is caused by AAPDs [43]. In light of this observation, a link between the gut microbiota, the production of peptide hormones, and the weight gain brought on by AAPDs may exist. An intriguing hypothesis regarding how intestinal bacteria can influence hormonal signaling pathways was presented by Fetissov and colleagues. In the blood of healthy individuals and rats, they discovered IgG and IgA autoantibodies specifically directed against leptin, ghrelin, PYY, neuropeptide Y, and other appetite-regulating hormones. These findings imply that the immune system affects the peptidergic system, which regulates hunger and emotions, as well as the microbiota that is connected with peptides, including *Bacteroides*, *Lactobacilli*, *Helicobacter pylori*, and *Candida* species [44].

For facilitating the reaction to strain in animals, the hypothalamic–pituitary–adrenal axis (HPA) system is the chief neuroendocrine system. This system is promoted by the release of vasopressin and corticotropin-releasing factor (CRF). The production of glucocorticoids is stimulated by the release of adrenocorticotropic hormone (ACTH) from the pituitary gland, which is encouraged by CRF and vasopressin. It is now known that the HPA axis malfunction significantly contributes to the emergence of anxiety and depression [45]. The microbiota in one’s gut can also affect the way the HPA axis works. According to a recent study, germ-free (GF) mice responded to moderate restraint stress by releasing corticosterone and ACTH more than normal. Colonization with the fecal microbiota of SPF animals was able to partially reverse this release, while monocolonization with *B. infantis* was able to fully restore it [46]. The SPF-stressed mice also showed substantially lower Fkbp5 transcription levels when GF animals and SPF-stressed mice underwent repeated social defeat procedures, which can enhance glucocorticoid receptor sensitivity and boost the effectiveness of the HPA axis negative feedback [47]. Another study found that the expression of behaviors resembling depression is decreased when genes associated with glucocorticoid receptors are upregulated in the hippocampus of GF mice [48]. According to a report, the bacterium *Faecalibacterium prausnitzii* ATCC 27766 has the potential to reduce the hyperreaction caused by CUMS on the HPA system and to increase the SCFAs in order to bring the inflammatory level down [49]. Intestinal dysfunction and microglial activation in the hippocampus were also corrected by *Clostridium butyricum Miyairi* 588 [50].

### 2.3. Gut Microbiota and Neurological Signaling

The flora of the digestive system is one of the most significant variables in the development of brain malformation [51]. The intestinal flora may affect the transcriptional activity of genes related to neuronal myelin [52], having the potential to bring about a change in the structural makeup of the brain that is long lasting. It was discovered through the utilization of various brain imaging technologies that alterations in the population of the gut microbiome may affect the integrity of the white matter [53]. In elderly Alzheimer’s patients, *Escherichia*/*Shigella* counts are up, whereas *E. rectale* counts are down [54]. Patients with major depressive disorder have been shown to have a surge in fecal *Bacteroidetes*, *Proteobacteria*, and *Actinobacteria*, as well as a decrease in fecal *Faecalibacterium*, all of which are associated with low levels of brain-derived neurotrophic factor (BDNF) in the serum [55]. In addition to this, it has been discovered that the prevalence of *Clostridium XIVb* has an inverse correlation with the level of BDNF in the blood [55]. Similarly, a study conducted on animals revealed that the schizophrenia-like behavior group has an increased prevalence of the genera *Roseburia, Dorea*, and *Odoribacter* [56]. Additionally, the newly published research shows that certain microbiota associated with schizophrenia, such as the family *Veillonellaceae*, has a positive relationship with the volume of the right middle frontal gyrus, while the regional grey matter is positively correlated with *Lachnospiraceae* and *Prevotellaceae* [57].

These alterations in the gut microbiota’s makeup are because of specific illness situations possibly act as a new diagnostic marker. The important plasticity-related protein BDNF is important for learning, memory, and emotional control. It is associated with neuron survival and supports neuron growth, development, and survival [58]. Brain-derived neurotrophic factor (BDNF) can be measured in the blood as a biomarker to represent its amount in the brain [59]. In addition, BDNF can lead to weight gain by increasing food intake while simultaneously decreasing energy consumption [60]. The gut microbiota regulates the expression of BDNF, which may cause gut cells to secrete BDNF. Overall, an unbalanced gut flora may change BDNF levels and trigger neuroinflammation, which is connected to the pathogenesis of obesity and dementia [61]. The exact ways that gut microbiota maintains BDNF, however, are mainly unidentified. In the central nervous system, the major inhibitory neurotransmitter known as GABA performs a crucial role in maintaining physiological and psychological homeostasis [62]. GABA production by *Bifidobacterium dentium* and *Lactobacillus brevis* has been demonstrated. Bacteria play a part in the production of GABA, and the resulting GABA can operate independently or as a secondary messenger to transmit signals from the vagal nerve to the enteric and central nervous systems [63,64]. In addition to its role as a signaling molecule for both the brain and the gut, serotonin (5-HT, also known as 5-hydroxytryptamine) plays a pivotal role in the signaling process that occurs along the brain–gut axis [65]. The peripheral nervous system and the brain both synthesize tryptophan, the only precursor to serotonin [66]. By controlling the metabolism of the kynurenine pathway, the microbiota in the gut such as *Streptococcus* spp., *Candida* spp., *Enterococcus* spp., and *Escherichia* spp. may alter serotonin production. This in turn affects both gastrointestinal and central nervous system function [67]. The enterochromatin cells in the gut produce the majority of the 5-HT in the body [38]. Through its effect on brown adipose tissue, peripheral serotonin is thought to have a significant bearing on the development of metabolic syndrome [68].

Several studies conducted on animals have found evidence that microorganisms in the gut may stimulate the vagus nerve, leading to signal transmission from the gut to the brain. Anorexia, lethargy, hyperalgesia, and a host of other brain processes and behaviors are all significantly regulated by this activation [63]. For instance, ingesting *L. rhamnosus JB1* alters the expression of genes that code for GABAergic receptors located in the amygdala and hippocampus, reducing anxiety-like behavior. These two brain regions are responsible for controlling anxiety and behavior [62]. However, when the vagus nerve was cut, the antidepressant effects of JB1 were no longer present. In another study, behavior and the expression of the BDNF gene were regulated through oral administration of *Bifidobacterium longum* [69]. According to the findings of these studies, the vagus nerve plays an extremely important part in the interaction that occurs between gut bacteria and the nervous system.

### 2.4. Crosstalk between Gut Microbiota and the Immuno-Inflammatory System

In the presence of inflammation, metabolic syndrome (MetS) is more strongly related to cognitive impairment [61]. In addition, Cuomo and colleagues emphasized that the gut microbiota influences immunity in one of three ways: either by activating the immune system; by secreting mediators; or by communicating with other mediators that can freely enter the brain [70], which activate the inflammatory pathway and have the potential to make metabolic syndrome and cognitive dysfunction worse. Innate fat cells and immune cells that have been activated have the potential to produce the proinflammatory cytokine IL-6 [71], which suggests that higher levels of body fat deposition are connected with higher levels of IL-6 production and a higher risk of cognitive impairment. The levels of three key pro-inflammatory mediators identified in the blood of obese patients, namely, IL-6, TNF-α, and CRP, are closely related to the participants’ waist circumference, weight, and body mass index [72]. According to the findings of one clinical trial, a higher level of IL-6 in older participants was related to a decreased abundance of *Ruminococcus* and *Prevotella*, together with an increased richness of the *Oscillibacter* co-abundance group [73]. Similarly, Biagi and associates showed a favorable association between the amount of circulating IL-6 and the number of bacteria in the genus *Proteobacteria*. This included *Escherichia coli* and its derivatives *Haemophilus*, *Pseudomonas*, *Klebsiella pneumoniae*, *Yersinia*, *Serratia*, and *Vibrio*. The bacteria *Eubacterium hallii et rel*., *Eubacterium rectale et rel*., and *Eubacterium ventriosum et rel*., as well as *Clostridium nexile et rel*. and species *Clostridium cluster XIVa*, were shown to have a negative connection with IL-6 levels [74].

TNF-α is involved in a wide variety of cellular processes due to its ability to both regulate and disrupt metabolic pathways, particularly those involved in lipid homeostasis [75]. TNF-α levels that are elevated beyond normal in the fatty and muscular tissues of obese humans may be responsible for the activation of multiple signal transduction cascades, which leads to an inflammatory response [76]. It was discovered that patients who took *Lactobacillus Plantarum P8* therapy for 12 weeks had lower levels of pro-inflammatory cytokines such as interferon-gamma and TNF-α when compared to patients who took a placebo. This finding is quite intriguing, along with the improvements found in memory and cognitive function. These improvements include social-affective cognition as well as verbal learning and memory [77]. In addition to this, it was discovered that *Odoribacter splanchnicus*, *Bilophila*, and *Bifidobacterium adolescentis* all had a negative correlation with the production of TNF-α [78]. According to OrbeOrihuela and colleagues, there is a positive association between TNF-α levels and the abundance of the phylum *Firmicutes* [79].

In the gut–brain connection, LPS plays a bidirectional communication [80]. Several studies were conducted to investigate the connections between the microbiota in the gut and LPS levels. The levels of circulating *Escherichia coli* were found to have a positive correlation with LPS levels in a study that included 64 people, 32 of whom were obese and 32 of whom had normal weights [81]. Another study found that increased bacterial translocation increases systemic exposure to LPS, which has been linked to MetS and cognitive problems via inflammatory responses [82,83]. LPS displacement from the colon to the portal vein, on the other hand, induces obesity-related low-grade inflammation in rats. This inflammation can be partially reversed by injecting the mice with bacteria that produce propionic acid, such as *Akkermansia muciniphil* [84].

#### 2.4.1. Gut Microbiota and Immune Pathways

Independently or in conjunction with one another, the immune system and the microbiota in the gut can regulate neurophysiology. Both innate and adaptive immune cells are abundant throughout the central nervous system [85]. It has been hypothesized that increased intestinal permeability, and possibly even blood–brain barrier (BBB) permeability, can lead to neurological diseases. Recent research has shown that certain extracellular pathogens, such as *Neisseria meningitidis*, *Escherichia coli*, and *Streptococcus*, are capable of invading host cells and causing disease. These pathogens have the potential to provoke a meningeal immune response that can affect social behavior, memory, and spatial learning [86]. A recent study found that an elevated level of the protein fatty-acid-binding protein-2 (FABP2) in those with depression or anxiety was a sign of intestinal barrier permeability [87]. The immune pathway that connects the microbiota in the gut to the central nervous system can travel in either direction. Alterations in the gut microbiota have the potential to cause shifts in the levels of pro- and anti-inflammatory cytokines that are circulating in the blood, and certain metabolites can directly affect CNS function. As a result, changes in brain biochemistry may result in changes in immunological responses and microbial composition via the HPA axis [88]. For instance, it has been shown that depressed mice exhibit a clear dysbiosis of the HPA axis as well as a high amount of inflammation in the central nervous system (with increased levels of TNF-α and IL-1 in the hippocampus) [89].

#### 2.4.2. Gut Microbiota and Inflammation

Neuschwander-Tetri and Caldwell found that there is growing evidence that directly links insulin resistance in the liver, muscle, and adipose tissue to the quantity of pro-inflammatory cytokines. These cytokines have multifaceted effects on the genes that are responsible for insulin resistance susceptibility, including those that regulate lipid synthesis, gluconeogenesis, and adipogenesis [90,91]. As a result, inflammation blocks insulin signaling pathways, which reduces the body’s sensitivity to insulin and increases the chance of developing insulin resistance [92,93]. Recent findings lend credence to the theory that shifts in the microbial flora of the gut and/or the metabolic activity of its inhabitants play a crucial role in the etiology of obesity and illnesses that are associated with it [94].

Lipopolysaccharide, a component of the cell wall of Gram-negative bacteria that reside in the gut, has been discovered as a significant role in the production of chronic inflammation that is metabolically driven and related to obesity [95]. An increase in the number of lipopolysaccharide producers and a drop in the proportion of intestinal barrier defenders showed that a high-fat diet had disrupted the gut microbiota. *Bifidobacterium* spp. releases lipopolysaccharide into the host’s circulation via a partially compromised intestinal barrier, acting as the main mediator of inflammation resulting in insulin resistance and obesity (metabolic endotoxemia) [95]. In obese mice fed a high-fat diet, the plasma concentration of lipopolysaccharide rose by two to three times. These outcomes are analogous to those observed in MetS-affected human individuals [96]. Oligofructose was added to help keep *bifidobacteria* at normal levels, which in turn helped keep the gut barrier less permeable to lipopolysaccharide. As a result, mice given a high-fat diet did not develop insulin resistance or obesity [97]. In human participants, it was discovered that a high-fat diet and an elevated body mass index (BMI) were related to higher lipopolysaccharide content [98,99]. Endotoxin-producing *Enterobacter* decreased from 35% to non-detectable levels in a morbidly obese volunteer’s gut bacteria after 23 weeks on a diet of whole grains, traditional Chinese medicinal foods, and prebiotics (WTP diet) [100].

**Figure 1:**
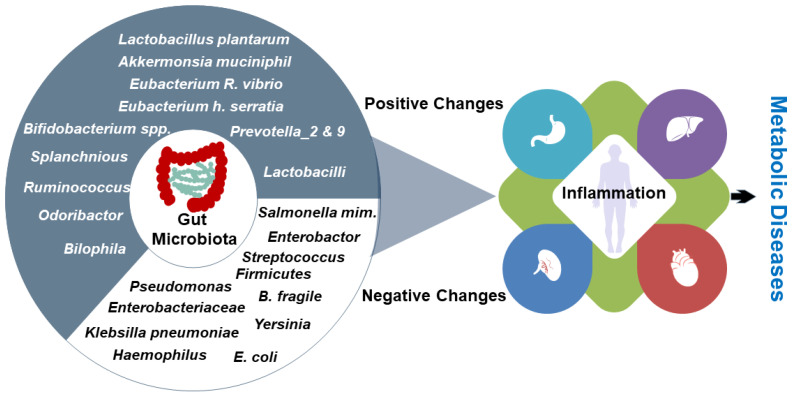
Inflammation-targeted gut microbiota.

Compared to *B. fragilis*-induced inflammation, *Enterobacteriaceae* causes a strong inflammatory response that is a thousand-fold larger [101,102]. Obesity-related changes in the gut microbiota have been linked to both local and systemic inflammation. For instance, plasma CRP levels were found to be elevated in these subjects and correlate with the ratio of *Bacteroidetes* to *Firmicutes* [103]. An inverse correlation between CRP concentrations and G+C abundance was observed. As a result, bacterial populations with high DNA GC concentrations might control inflammatory reactions in the host [104]. Intervention with high levels of cocoa flavonol in healthy human volunteers led to a significant reduction in CRP concentrations, which correlated with the levels of *Bifidobacteria* and *Lactobacilli* [105]. In conclusion, there is a significant abundance of microbes associated with the nutrigenomic approach boosting the immune system and regulating inflammation in metabolic diseases. At the same time during abnormal conditions such as inflammatory diseases, some other microbes trigger a large number of metabolic and signaling pathways in different tissues, which contribute to metabolic diseases (Figure 1).

We identified specific gut microbiota that target inflammation associated with metabolic diseases in different parts of the body. This specific microbiota should be targeted for personalized diagnosis and nutrition therapy of metabolic diseases to reduce the development and progression of inflammation. The blue part indicates positive changes and the white part negative changes in microbiota-associated inflammation.

## 3. Time-Restricted Feeding/Eating: A Targeted Approach

The host physiology, environment, and daily dietary changes all play a role in gut microbiota homeostasis [106]. The make-up and/or activity of gut microbiota is a factor that distinguishes individuals who are obese from those who are lean, as well as diabetic patients from those who do not have diabetes [107,108,109]. What is more important is that the alterations in gut microbes that are associated with the aforementioned disorders can be reversed by nutritional intervention [108,110]. This is because of the preeminent role that diet and the timing of eating play in shaping the composition of the gut microbiota as well as the gene transcription network [111,112]. A promising strategy for the management of obesity and metabolic diseases is the modification of gut microbiota through the eating pattern and consumption of nutrients that contain prebiotic properties [113,114]. This eating plan ought to not only fulfill the dietary requirements of human beings but also maintain a healthy microbiota in the gut. A diet that prevents MetS should be high in whole grains, fruits, vegetables, lean meats and fish, and low-fat or fat-free dairy products and low in processed foods, which may contribute to a diverse microbial flora [115].

The manipulation of the gut microbiota composition through dietary changes and intermittent fasting (IF) has emerged as a potentially effective “pharmaco-nutritional” strategy for reversing dysbiosis and host metabolic disorders [116,117]. However, the conventional medical care system does not yet have the capability of evaluating both the qualitative and quantitative changes that occur in the gut microbiota. At the population level, one potential strategy for the prevention and management of metabolic syndrome should involve the development of a set of approaches related to changes in the microbiota of the gut. TRF stands for time-restricted feeding in animals and time-restricted eating (TRE) in humans throughout a counted number of hours. It allows for a daily fasting duration that is greater than 12 h, and it does so without affecting either the quality or quantity of the nutrients consumed [118]. Through the involvement of circadian genes and the gut microbiome, time-restricted feeding/eating (TRF/E) provides protection against nutritional challenges that can lead to obesity and metabolic risks [8]. It has been hypothesized that TRF/E may regulate and modulate gut microbiota in order to prevent metabolic disease through multiple pathways.

### 3.1. Communication between TRF/E and Gut Microbiota

It is still too soon to determine how TRF/E affects the composition of the gut and the functions it performs through daily feeding and fasting rhythms. These daily rhythms in gut physiology provide context and a basis for adopting TRF to maintain gut health. Lean meats and fish, fruits, vegetables, whole grains, and low-fat or fat-free dairy products should all be abundant in a diet that reduces MetS. The variety of the gut microbiota is increased by these modifications. Therefore, the feeding pattern and duration, in addition to the composition of the diet, are important parameters to consider when determining the contribution of the microbiome to the physiology and the host nutritional status [21]. Communication between the gut and the brain is essential for determining the appropriate portion size of a meal and sending signals to the brain to control feelings of hunger and fullness. It has been reported that TRF resulting from the same obesogenic diet can restore the daily rhythm of GVA responsiveness to meal size [119].

Previously, TRF imposed significant alteration in the microbial composition of human gut microbiota (Table 1). There were substantial alterations and relative richness of bacterial communities in healthy persons using combined effect size measures from linear discriminant analysis (LDA). These communities were classified as either TRF or non-TRF. At the level of the genus, 34 bacteria were enriched in the TRF group, and 18 bacteria were enriched in the non-TRF group. The most numerous genera in the TRF group were *Bacteroidetes* and *Prevotellaceae* (prevotella 9 and prevotella 2), while the most numerous genera in the non-TRF group were *Escherichia*, *Shigella*, and *Peptostreptococcus* [9]. Similarly, a study revealed that timed-feeding protocols (TRF, alternate day fasting and caloric restriction) induced measurable sifts in the bacterial compositions in mice that coincide with improvements in metabolism [120]. TRF, on the other hand, was successful in reestablishing cyclical variation in several bacterial families that are thought to play a role in metabolism [21]. In the *Lactobacillus* family, TRF was able to restore cyclical variation, which is likewise cyclical in regular chow animals but not in DIO mice. Diabetes and obesity have been linked to a number of different species of the genus *Lactobacillus* [121,122,123]. *Lactobacillus* species express bile salt hydrolases, which are responsible for the conjugation of gut luminal bile acids (BAs), and they have the ability to affect BA signaling [21]. In addition, TRF was successful in reintroducing members of the *Ruminococcacea* family, such as those belonging to the genus *Oscillibacter*, which are thought to provide resistance to the metabolic effects of obesity [123]. A larger number of *Firmicutes* species in the gut microbiome has been associated with increased adiposity, suggesting that the *Firmicutes* phylum may play a role in the development of obesity. According to the results of research that evaluated the microbiome at several time periods in normal mice as well as in TRF mice, the amount of *Firmicutes* species is connected to the food and feeding pattern rather than obesity or dysmetabolism itself. The *Firmicutes* phylum, as a whole, is not obesogenic, and it may alter within 24 h after a change in diet [124,125]. In addition to that, it was hypothesized that having a low alpha diversity in the gut microbiome was also a contributor to obesity. However, when the alpha diversity was averaged between all of the different time points, there was no difference between the mice that were fed normal chow ad libitum, TRF, or DIO. Contrary to the metabolic phenotype, fluctuations in alpha diversity were found to be related to diet and the amount of time feeding [21]. Another study demonstrated that 12 weeks of TRF did not significantly alter the diversity or overall composition of gut microbiome in adults with obesity [126].

It is well acknowledged that having a variety of species residing in the gut microflora protects against metabolic illnesses and obesity. The results of metabolomics studies performed on the feces of mice that had been fed ad libitum with TRF revealed significant differences, which may help to explain some of the improvements observed in the TRF mice. Hemicellulose found in food is typically decomposed into xylose and galactose by the microbes that live in the gut, and the host can absorb a portion of this. The fact that TRF mice excreted a significantly higher amount of xylose and galactose in their stool compared to ad libitum-fed mice suggests that TRF lowered the amount of these simple sugars that were absorbed by the host. Both primary and secondary bile acids were found in high concentrations in the feces of TRF mice. This suggests that TRF facilitates the reabsorption of bile acids from the gastrointestinal tract. The fact that there were lower levels of bile acids in the stool may be responsible for at least some of the decrease in hepatic and serum cholesterol that was observed in TRF mice [21]. TRF is associated with better metabolic health, perhaps owing to changes in gut microbiome and circadian pattern of molecules related to liver metabolism. However, a previous study demonstrated that TRF showed distinct circadian rhythms in liver expression of PPARα, SREBP, and Sirt1 as well as the circadian rhythm of the abundance of *Bacteroidetes* and *Firmicutes* [134].

### 3.2. TRF/E and Circadian Rhythm

The changes in the levels or activities of nicotinamide adenine dinucleotide (NAD) and sirtuins, depending on the energy state of the cell, affect the circadian clock [135,136]. However, AMPK phosphorylates CRY and encourages breakdown of the energy state of the cell during fasting that affects the circadian system [137]. As a result, the presence of eating and fasting cycles enhances the robustness or amplitude of the oscillation of circadian activator and repressor components. The lack of a functioning circadian clock can result in some oscillations in transcription, downstream metabolites, and even the gut microbiota [124]. However, these signals cannot completely make up for the loss of the circadian clock. To guarantee that anabolic and catabolic forms of metabolism are coordinately controlled in line with the activity/rest cycle, the circadian oscillation and feeding/fasting signals combine synergistically. This is accomplished by ensuring that the circadian oscillator is in sync with the feeding/fasting signals. TRF may adjust the phase of peripheral oscillators to make them coincide with the phase of the central oscillations. Through hormonal synchrony, intermittent fasting (IF) can have an effect on the circadian rhythmicity. Early in the morning, the circadian insulin secretion reaches its highest point, then continuing to rise throughout and after meals [138,139]. Performing TRF daily in the morning reduces insulin levels not only after meals but also for an average of 24 h, which ultimately increases insulin sensitivity [48,140].

**Figure 2:**
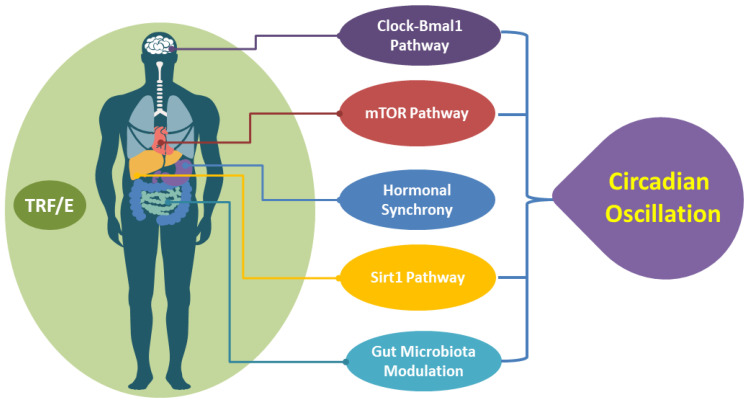
TRF/E-targeted pathways for the oscillation of the circadian rhythm.

TRF can alter circadian-driven processes due to downstream effects caused by an inhibited mTOR pathway [137,141]. The phosphorylated kinases (AMPK, CK1, and GSK3) that are activated by mTOR play a direct role in regulating the expression of CRY1 and CRY2 during times of fasting. Similarly, the mTOR pathway is responsible for the increased circadian phosphorylation of CREB, which can activate PER (period circadian protein) transcription [142]. The practice of TRF affects circadian rhythmicity through these mechanisms, which can lead to coupled and strengthened peripheral and central genes, hormones, and protein secretion [141]. As a result, TRF is responsible for optimal rhythms of behavior, physiology, gut microbiota, molecular pathways, and metabolism, and it ensures harmony with an individual’s activity/rest cycle and health span (Figure 2). Recent research has shown that TRF is responsible for regulating the circadian rhythm and its stimulators in humans, which is necessary for metabolic health. TRF intervention resulted in a significant increase in the level of mRNA expression of the Bmal1 gene (*p* = 0.0020) and the Clock gene (*p* = 0.0302) [8]. It is possible that activation of Sirt1 can also modulate mice’s circadian physiology [143]. Additionally, our results demonstrated that the activation of Sirt1 can control the circadian rhythm. The mRNA level of Sirt1 was significantly upregulated, just as it was in the post-TRF group, in comparison to the pre-TRF group and the non-TRF group, respectively [8].

This figure shows that TRE may target many cellular and genetic pathways that contribute to the alignment of circadian rhythm with host metabolism. TRE may modulate the Clock–Bmal1 pathway, synchronize hormonal signals, regulate the Sirt1 pathway, inhibit mTOR signaling, and modulate gut-microbiome-related nutrient sensors.

Recent studies have shown that the emergence of obesity is correlated with reduced microbiome diversity, changed gut microbial activity, and dispersed microbiome relative abundance, especially of two phyla, namely, *Bacteroidetes* and *Firmicutes* [23]. A microbial community’s complexity may be seen in its microbial richness, which is a gauge of alpha diversity for the gut microbiota. A more diverse gut microbiota is linked to better health [144,145]. According to Sonnenburg and Backhed (2016), the gut microbiota may be able to influence systemic metabolic responses [146]. According to earlier studies, Sirt1 is a key regulator and promoter of the expression of clock genes and acts as a molecular bridge between circadian rhythms and metabolic regulation [18]. A study revealed that NADH cycle in liver links the nutrients state to whole body energetics through the circadian regulation of Sirt1 [139]. More interestingly, a previous study demonstrated that early TRE improves 24-hour glucose levels and changes circadian clock gene expression and lipid metabolism. However, there is increased autophagy due to the anti-aging effect of early TRE in humans [147]. This represents the fact that TRE may regulate the circadian oscillation for a healthy lifespan.

### 3.3. TRF/E and Metabolic Regulators

The level of glucose in the blood drops during intermittent fasting, and as a result of the process of lipolysis, the fats in the body (triacylglycerols and diacylglycerols) are broken down into free fatty acids (FFAs). Then, these lipids are transported to the liver, where they undergo oxidation and proceed through the intermediary steps of acetyl CoA and HMG-CoA before becoming ketones (acetoacetate (AcAc) and hydroxybutyrate (BHB)). Both BHB and AcAc are brought from the blood into the brain, where they are eventually taken up by neurons. Aside from the metabolic process of ketone bodies that occurs in the liver, astrocytes are also capable of the process of ketogenesis, which may serve as an important local source of BHB for neurons. Due to a decrease in the amount of glucose that is readily available and an increase in the number of ketones, the ratio of AMP to ATP in neurons is decreased. The kinases AMPK and CaKMII are activated as a consequence, and CREB and PGC1 are consequently activated that, in turn, stimulate autophagy. BHB can increase the expression of brain-derived neurotropic factor (BDNF), which may support mitochondrial biogenesis, synaptic plasticity, and cellular stress tolerance. On the other hand, IF causes a reduction in the amount of insulin that is circulating in the blood, which boosts neuroplasticity and protects against metabolic and oxidative stress via the insulin/IGF signaling pathway [148].

The circadian clock oscillations; the cycling of metabolic regulators such as CREB (cAMP response element-binding protein), AMPK, and mTOR; and the expression of their target genes are all restored by TRF [137]. The nuclear factors PPARg (peroxisome proliferator-activated receptor gamma) and PGC-1 alpha (peroxisome proliferator-activated receptor gamma coactivator 1-alpha), which have several metabolic effects, are modulated by TRF, which controls the levels of SIRT1. TRF also has a promising effect on nicotinamide adenine dinucleotide (NAD+) [149]. The studies conducted on rodents showed that decreasing the daily eating window has striking effects on metabolism, body weight, and composition with increased oxidation of fat and energy expenditure [150]. TRF has promising health roles as it not only improves cardiometabolic health and reduces weight but can also slow down the progression of the tumor, delay the process of aging, and eventually increase lifespan through the execution of signaling pathways. The presence of SIRT1 suggests that it promotes longevity via protection against DNA damage. Due to increased metabolic roles, it lowers the insulin levels and fasting glucose in the morning, with the increased production of insulin in the evening, which leads to the decreased 24-hour glycemic index. An increase in fat oxidation due to prolonged fasting periods in a day leads to higher levels of LDL and HDL [151,152]. Early TRE can improve insulin sensitivity, blood pressure, and beta cell function through stimulation of insulin [153]. Gut microbiota may be able to regulate systemic metabolic responses, and TRF can regulate the gut microbiota, which in turn regulates the genetic pathways [146]. Therefore, we observed in a recent study that Sirt1 expression and serum HDL showed a positive correlation with gut microbiome richness in the TRF group. This suggests that TRF lessens the burden of metabolic risk by regulating Sirt1 expression and serum HDL levels in response to modulation of the gut microbiome. Therefore, compared to the group that did not receive TRF, the TRF group had a significantly higher microbial diversity [8].

The treatment of metabolic diseases in modern humans presents several difficult medical challenges. Intermittent fasting is a therapeutic lifestyle strategy that can lower the risk of various metabolic illnesses, including obesity and hypertension [154]. TRF alters the levels of lipids, metabolic regulators, and inflammatory cytokines in the body to reverse and prevent diet-induced obesity (DIO) and related metabolic disorders in animals and humans without changing dietary composition. This is accomplished in the absence of any changes in dietary intake [155]. We looked into whether or not TRF affects lowering hyperlipidemia in humans. TRF resulted in a significant reduction in serum levels of both total cholesterol and triglycerides while elevating HDL levels. Regular consumption of large amounts of energy at all hours of the day and night has been linked unquestionably to the development of obesity and, ultimately, to the disruption of liver enzymes. Our study revealed significant decreases in the levels of the alkaline phosphatase/-glutamyl transferase, aspartate aminotransferase, and alanine aminotransferase [8].

### 3.4. TRF/E and Inflammatory Signaling

Even in COVID-19 infection, inflammation plays a pivotal role in the development of insulin resistance and cytokine release syndrome. This is because different cytokines can influence a wide variety of molecular pathways. Insulin resistance, for instance, may be induced by TNF-α via the JNK and IKK/NF-B (jun amino-terminal kinase/inhibitor of NF- kinase) pathways, which may lead to an increase in the serine/threonine phosphorylation of insulin receptor substrate 1. In addition, IL-6 has the potential to reduce insulin sensitivity in skeletal muscle through the induction of toll-like receptor-4 (TLR-4) gene expression via the activation of STAT3, which is an activator of transcription 3. The activation of IKK/NF-B signaling could, in turn, stimulate the production of TNF-α, indicating that this relationship is potentially two-way [156]. TNF-α and IL-1 levels were shown to be lower in the TRF group than in the normal diet (ND) group, whereas IL-6 levels seemed to drop in the TRF group but were not statistically different from ND. TRF was found to modulate some of these inflammatory markers, and it was seen that IL-6 decreased in the TRF group [157]. Previous information on the effect of IF on inflammatory markers is scant, but the results of our most recent investigation suggested that TRE lowered the production of pro-inflammatory cytokines [8]. Excessive consumption of energy-dense food will result in the production of an inflammatory response, which is a causal factor in the dysregulation of glucose and lipid metabolism [158]. Dyslipidemia as well as inflammation linked to obesity can contribute to the development of atherosclerosis, the clinical manifestation of vascular inflammation in metabolic disorders [159]. Concerning the part that inflammation plays in the development of atherosclerosis, the level of IL-1 is elevated in atherosclerosis and is related to the severity of the disease [160]. TNF-α and IL-1 are the most important pro-inflammatory cytokines that can be traced back to metabolic dysregulation. These cytokines are secreted by adipose tissue [161]. Despite this, we discovered that the post-TRF group had much lower blood and mRNA levels of the cytokines IL-1 and TNF-α than the pre-TRF group and the non-TRF group; however, this difference did not reach statistical significance [8]. This is the significant effect of TRF on inflammation to reduce the burden of chronic diseases through these mechanisms.

### 3.5. TRF/E and Hormonal Signaling

A great number of hormones can be thought of as nutritional signals, and the receptors on their ends play critical roles in mediating the effects of nutrition on a large number of genes that are involved in growth, metabolism, and signaling pathways. According to the findings of a previous study, the levels of total testosterone and IGF-1 in TRF significantly dropped after 8 weeks of intervention with TRE. Only in TRE subjects, there was a significant reduction in blood glucose and insulin levels, and in line with this, a significant improvement in HOMA-IR was found. Within the TRF group, there was a significant increase in adiponectin and a significant decrease in leptin and T3; however, there was no significant change in TSH [157]. As a result, TRF mice showed an increase in adiponectin and a decrease in leptin [42,162]. In another study, the TSH was measured to look for signs of thyroid dysfunction, which could influence other metabolic endpoints. They noted a tendency toward an increase in TSH with 10 h of TRE intervention [163].

## 4. Conclusions

Gut microbiota dysbiosis (low abundance) is associated with enhanced development of inflammatory diseases, obesity, atherosclerosis, neurodegenerative diseases, and diabetes. Therefore, many underlining factors of metabolic diseases are reversed or improved by gut microbiota modulation through TRF/E. Some specific microbiota such as *Lactobacillus Plantarum P8* improve cognitive and memory function; *L. Rhamnosus JB1* decreases anxiety and controls fear and emotions; *Clostridium butyricum* restores intestinal dysfunction and hippocampal microglial activation; *Faecalibacterium prausnitzii* decreases inflammation; *Oscillibacter* spp. and *Lactobacillus* spp. regulate the host metabolism via glucose and lipid metabolism; *Lactobacillus* and *Bifidobacterium* help to maintain weight; *Prevotella* facilitates peripheral clock adjustment; *Prevotellaceae*, *Bacteroidia*, and *Dialisster* control the circadian system that regulates intestinal physiology and systemic metabolism; *Prevotella* and *Bacteroidia* improve circadian rhythmicity; *Bacteroidia* exhibits an anti-obesity response (Table 2). These indicate that specific gut microbiota can be targeted as biomarkers because of their involvement in many biological, cellular, and metabolic processes. On the other hand, an approach in the form of TRF/E is essential in terms of target nutrient utilization and host nutritional status metabolism through modulation of the gut microbiota and the circadian system. This approach showed an extensive effect in the recovery of gut microbiota dysbiosis. However, TRF/E may contribute to the prevention of metabolic diseases via modulation of the Clock–Bmal1 pathway, synchronizing hormonal signals, regulating the Sirt1 pathway, inhibiting mTOR signaling, and modulating gut-microbiome-related nutrient-sensors.

## Figures and Tables

**Table 1:** TRF-induced changes in the gut microbiome: evidence from human studies.

Fasting Hours and Duration of TRF	Number of Subjects	Changes in Gut Microbiome	Sequencing Scheme	Reference
16 h/25 days	80 healthy male adults	*↑* microbial diversity *↑* abundance of *Bacteroidetes* and *Prevotellaceae*	16s rRNA (ribosomal ribonucleic acid)	[8]
8 h/12 weeks	14 adults with obesity	No significant changes in the abundance of microbiota	16s rRNA	[126]
12 h/12 weeks	24 patients with obesity	*↑* in the frequency of *Lachnospiraceae*, *Parasutterella*, and *Romboutsia*	16s rRNA	[127]
2 day modified IF/8 weeks	39 patients with metabolic syndrome	Induced significant changes in gut microbiota communities *↑* production of short-chain fatty acids ↓ circulating levels of lipopolysaccharides	16S rRNA sequencing	[128]
R-TRF/4 weeks	30 healthy male adults	*↑* microbial diversity and remodeling of microbiome composition Provoked upregulation of butyric-acid-producing *Lachnospiraceae*	16S rRNA sequencing	[129]
R-TRF/4 weeks	34 healthy adults	*↑* alpha and beta diversity *↑* abundance of *Prevotella*, *Faecalibacterium*, Bacteroidetes, and Firmicutes	16S rRNA sequencing	[130]
16 h/26 days	45 healthy young adults	*↑* alpha diversity *↑* anti-inflammatory bacteria *Lactobacillus* and *Bifidobacterium* ↓ pathogenic bacteria	16S rRNA sequencing	[131]
R-TRF/29 days	9 healthy adults	*↑* microbial richness Enriched genera including *Butyricicoccus*, *Bacteroides*, *Faecalibacterium*, *Roseburia*, *Allobaculum*, *Eubacterium*, *Dialister*, and *Erysipelotrichi*	16S rRNA	[132]
17 h/29 days	9 healthy adults	*↑* abundance of healthy gut microbiota members (*Akkermansia muciniphila*, *Faecalibacterium prausnitzii*, *Bifidobacterium* spp., *Lactobacillus* spp., *Bacteroides fragilis* group, and *Enterobacteriaceae*)	16S rRNA	[133]
16 h e-TRF and m-TRF/5 weeks	82 healthy individuals without obesity	*↑* gut microbial diversity	16S rDNA	[134]

↑ *=* increase, ↓ = decrease, rRNA = ribosomal ribonucleic acid, R-TRF = Ramadan time-restricted feeding, e-TRF = early time-restricted feeding, m-TRF = mid-day time restricted feeding.

**Table 2:** Gut microbiota and their targeted mechanisms and actions.

Gut Microbiome	Target Abundance/Mechanism	Action	Reference
*Bacteriodetes*	Low abundance	Enhanced the development of inflammatory conditions, obesity, atherosclerosis, neurodegenerative diseases, and diabetes	[25]
*Bacteroidia*	Inversely correlated with LDL-c and triglyceride level	Exhibited anti-obesity response	[26]
*Bacteriodetes*	Increased abundance	Directly associated with weight loss	[26]
*Prevotella* and *Bacteroidia*	Significant positive correlation with Bmal1	Improved circadian rhythmicity	[8]
*Prevotellaceae*, *Bacteroidia*, and *Dialisster*	Positive correlation with Sirt1	Controlled the circadian system that regulate intestinal physiology and systemic metabolism	[8,28]
*Prevotella*	Produced SCFAs	Facilitated peripheral clock adjustment	[29]
*Lactobacillus* and *Bifdobacterium*	Modulated the GHS-R1a receptor to influence the ghrelin system	Helped to maintain weight loss in AAPDs-induced overweight patients	[36]
*Oscillibacter* spp. and *Lactobacillus* spp.	Helped in releasing glucagon-like peptide-1 (GLP-1) and peptide YY (PYY) hormones	Regulated host metabolism via glucose and lipid metabolism	[38,39]
*Bacteroides*, *Lactobacilli*, *Helicobacter pylori*, *Candida specie*, and *Escherichia coli*	Change in appetite and emotion-controlled peptidergic system	Interfered with the immune system	[44]
*Faecalibacterium prausnitzii*	Alleviated CUMS, induce HPA axis hyper reaction, and upregulate the SCFAs	Decreased the inflammatory level	[89]
*Clostridium butyricum*	Enhanced SCFA production	Restored intestinal dysfunction and hippocampal microglial activation	[50]
*Lactobacillus brevis* and *Bifdobacterium dentium*	Produced GABA neurotransmitters	Modulated physiological and psychological processes in the central nervous system	[64]
*Streptococcus* spp., *Candida* spp., *Enterococcus* spp., and *Escherichia* spp.	Affected tryptophan metabolism and subsequent serotonin synthesis by regulating the kynurenine metabolism pathway	Influenced cognition function in central areas as well as gastrointestinal function	[67,163]
*L. rhamnosus* JB1	Altered the expression of genes encoding GABA receptors in the amygdala and hippocampus	Decreased anxiety-like behavior, controlling fear and emotions	[62]
*Ruminococcus* and *Prevotella*	Low abundance	Associated with an increased level of IL-6	[73]
*Lactobacillus Plantarum* P8	Decreased pro-inflammatory cytokines, such as interferon-gamma and TNF-α	Improved memory and cognitive function	[77]
*Odoribacter splanchnicus*, the *Bilophila*, and *Bifdobacterium adolescentis*	Negatively correlated with TNF-α production	Regulated the inflammation process	[78]
*Neisseria meningitidis*, *Escherichia coli*, and *Streptococcus*	Induced a meningeal immune response	Affected spatial learning, memory, and social behavior	[86]
*Bacteroidetes*/*Firmicutes* ratio	Increased plasma CRP	Associated with local and systemic inflammation in obesity	[103]

LDL-C = low-density lipoprotein cholesterol, Sirt1 = Sirtuin 1, SCFAs = short-chain fatty acids, GHS-R1a = growth hormone secretagogue receptor type 1a, CUMS = chronic unpredictable mild stress, HPA = hypothalamic–pituitary–adrenal, GABA = gamma-aminobutyric acid, TNF-α = tumor necrosis factor-alpha, CRP = C-reactive protein.

## Data Availability

Not applicable.
